# Effect of Dietary Medium-Chain α-Monoglycerides on the Growth Performance, Intestinal Histomorphology, Amino Acid Digestibility, and Broiler Chickens’ Blood Biochemical Parameters

**DOI:** 10.3390/ani11010057

**Published:** 2020-12-30

**Authors:** Shimaa A. Amer, Afaf A-Nasser, Hanan S. Al-Khalaifah, Dina M. M. AlSadek, Doaa M. Abdel fattah, Elshimaa M. Roushdy, Wafaa R. I. A. Sherief, Mohamed F. M. Farag, Dalia E. Altohamy, Ahmed A. A. Abdel-Wareth, Abdallah E. Metwally

**Affiliations:** 1 Department of Nutrition and Clinical Nutrition, Faculty of Veterinary Medicine, Zagazig University, Zagazig 44511, Egypt; drabdalla75@yahoo.com; 2 Environment and Life Sciences Research Center, Kuwait Institute for Scientific Research, P.O. Box 24885, Safat 13109, Kuwait; drhanan14@gmail.com (A.A.-N.); hkhalifa@kisr.edu.kw (H.S.A.-K.); 3 Department of Histology and Cytology, Faculty of Veterinary Medicine, Zagazig University, Zagazig 44511, Egypt; dinaaalsadek@yahoo.com; 4 Department of Biochemistry, Faculty of Veterinary Medicine, Zagazig University, Zagazig 44511, Egypt; drdoaa30@yahoo.com; 5 Animal Wealth Development Department, Faculty of Veterinary Medicine, Zagazig University, Zagazig 44511, Egypt; Shimaa_production@yahoo.com (E.M.R.); Wafaa.production@yahoo.com (W.R.I.A.S.); 6 Department of Clinical Pathology, Faculty of Veterinary Medicine, Zagazig University, Zagazig 44511, Egypt; farag_cell@yahoo.com; 7 Department of Pharmacology, Central Laboratory, Faculty of Veterinary Medicine, Zagazig University, Zagazig 44511, Egypt; daliaaram1975@gmail.com; 8 Department of Animal and Poultry Production, Faculty of Agriculture, South Valley University, Qena 83523, Egypt; A.wareth@agr.svu.edu.eg

**Keywords:** broiler chickens, glycerol monolaurate, growth performance, amino acid digestibility, gut health

## Abstract

**Simple Summary:**

The addition of biologically active materials to animal feed is a very recent topic regarding antibiotic alternatives. This study inspected the influence of graded levels of medium-chain α-monoglycerides, glycerol monolaurate (GML) on the growth performance, apparent ileal digestibility coefficient (AID%) of amino acids, and intestinal histomorphology of broiler chickens. Broiler chickens (76.82 g ± 0.40, *n* = 200) were fed on four experimental diets that were complemented with 0; 1; 3; or 5 g kg^−1^ glycerol monolaurate (GML0; GML1; GML3; and GML5). The findings suggested that glycerol monolaurate supplementation can improve the immune status and intestinal histomorphology of broiler chickens with no improving effect on the growth performance.

**Abstract:**

This trial was conducted to assess the impact of medium-chain α-monoglycerides, glycerol monolaurate (GML) supplementation on the growth performance, apparent ileal digestibility coefficient (AID%) of amino acids, intestinal histomorphology, and blood biochemical parameters of broiler chickens. Three-day-old chicks (76.82 g ± 0.40, *n* = 200) were haphazardly allocated to four experimental groups with five replicates for each (10 chicks/replicate). The treatments consisted of basal diets supplemented with four glycerol monolaurate levels; 0, 1, 3, or 5 g kg^−1^ (GML0, GML1, GML3, and GML5, respectively). Growth performance was determined at three periods (starter, grower, and finisher). Dietary GML had no significant effect on the growth performance parameters (body weight, weight gain, and feed conversion ratio) through all the experimental periods. GML1 diet increased the AID% of leucine and decreased the AID% of arginine. GML1 diet increased the duodenal and jejunal villous height and the jejunal muscle thickness. GML3 and GML5 diets increased the goblet cell count in the duodenum. GML supplementation increased the serum level of high density lipoprotein (HDL)-cholesterol. GML5 diet increased the serum levels of IgM and interleukin 10 compared to the control group. We could conclude that dietary supplementation of glycerol monolaurate can supplement broiler chicken diets up to 5 g kg^−1^ to enhance the immune status and intestinal histomorphology of birds with no improving effect on growth performance.

## 1. Introduction

The replacement of antibiotics with biologically active materials in animal feed is a very recent topic. Modern feed production relies on the addition of bioactive constituents to the nutrition, which would decrease the content of antibiotics and other drugs, and have a positive effect on animal health and well-being. These components also reduce the negative effect of environmental stressors on the immune systems and production standards of an animal. Thus, in animal nutrition, the focus is on competitive exclusion, antibacterial peptides, prebiotics, probiotics, yeasts, and other additives [1,2,3]. These additives, which can be supplemented to chickens and piglets feed, are medium-chain fatty acids (MCFAs). Different MCFA marketable products are now accessible in the marketplace [4,5].

Medium Chain Fatty Acids, 6–12 carbonic saturated fatty acids, are obviously present as medium-chain triglycerides (MCTs) in milk fats and many nutrients, particularly palm oils, coconut, and cobia seed oils [6,7,8]. This group involves hexanoic acid (C6:0), octanoic acid (C8:0), and decanoic acid (C10:0). Lauric acid (dodecanoic acid, C12:0) is also classified with the MCFAs [9]. Both MCTs and MCFAs have definite metabolic and nutritional effects that are important in feeding young animals, including improved digestion, passive absorption, and obligatory oxidation [10]. Hydrolysis of MCTs occurs quickly compared to long-chain triglycerides (LCTs) without the need for emulsion with bile because of its high solubility in water.

MCTs are initially digested in the stomach by lingual and gastric lipases producing substantial MCFAs and monoglycerides, before entering the small intestine. In the duodenum, pancreatic lipases are released; the absorption of MCFAs becomes available [11]. Most MCFAs are absorbed in their free form by passive diffusion, but absorption as acyl ester has also been established [12]. MCFAs are not re-esterified within the intestinal cells but are diffused into the portal blood due to a low fatty acid-binding protein affinity, linked to albumin, and transported directly to the liver [13,14,15]. A minor proportion of MCFAs are ingested by chylomicrons [9,16]. Moreover, MCFAs have an antibacterial activity like short-chain fatty acids [17].

In poultry production, MCFA and related glycerides have apparent effects on production performance and egg quality [18,19]. Medium-chain α-monoglycerides are auspicious feed additives for the broilers industry. Glycerol monolaurate (GML) made from lauric acid and glycerol that lacks of toxicity, exhibits growth promoter capacity and potent antimicrobial activity [20]. They suggested that MGL could be used as an alternative antimicrobial in poultry farming. The current study aimed to evaluate the impact of dietary supplementation of glycerol monolaurate on the growth performance, amino acid ileal digestibility, blood biochemical parameters, and intestinal histology of broiler chickens.

## 2. Materials and Methods

### 2.1. Birds, Experimental Design, and Diets

The ethics of the experimental protocol were approved by the Institutional Animal Care and Use Committee of Zagazig University, Egypt (ZUIACUC–2019). All animal experiments were performed following the recommendations described in “The Guide for the Care and Use of Laboratory Animals in scientific investigations.”

Two hundred one-day-old chicks (Ross 308 broiler) were obtained from a marketable chick producer. Before starting the experiment, chicks were submitted to a 3-day adaptation period to reach an initial body weight of 76.82 g ± 0.40. They were haphazardly allocated to four experimental groups with five replicates (10 chicks/replicate). Birds were fed on basal diets supplemented with four levels of glycerol monolaurate: 0, 1, 3, or 5 g kg^−1^ (GML0, GML1, GML3, and GML5, respectively) (glycerol monolaurate, FRA^®^ C12, Framelco, Ruisvoorn 5, 4941 SB, Raamsdonksveer, Holanda). The experiment lasted for 35 days, with continuous lighting and adequate ventilation. Freshwater and feed were offered for ad libitum consumption throughout the investigation. The routine health and vaccination practices were implemented strictly according to the recommendations. The chicks were checked daily to ensure there were no health problems. The formula and chemical composition of the basal diet are shown in (Table 1). An approximate chemical analysis of the feed used and experimental diets was performed according to the standard procedures for the Ross 308 broiler feed specification, AVIAGEN [21].

### 2.2. Growth Performance

The average initial body weight was obtained on the 4th day of age. Then the body weight was recorded at 10, 23, 35 days.

The body weight gain (g/bird) = W2 − W1, where W2 is the final body weight at the intended period, and W1 is the initial body weight in the same period.

Feed intake (g/bird) = feed offered weight − residues left/birds No.

The feed conversion ratio (FCR) was estimated weekly: FCR = the amount of feed consumed (g)/Bodyweight gain (g).

The relative growth rate (RGR) was calculated using the equation described by [22].

RGR = W2 − W1**/**½ (W1 + W2) × 100. W1 = the initial live weight (g), W2 = the live weight at the end of the considered period (g).

Protein efficiency ratio (PER) was determined according to [23].

PER = Live weight gain (g)/Protein intake (g).

### 2.3. Amino Acids Ileal Digestibility

Titanium dioxide, an indigestible indicator substance, was used to determine the amino acid ileal digestibility described by [24]. The amino acid concentration in the diet and ileal digesta samples were measured according to Li et al. [25,26]. Tryptophan was measured separately, according to Ravindran and Bryden [27]. Titanium dioxide was valued following the procedures of Fenton and Fenton [28]. The apparent ileal digestibility coefficient (AID%) of amino acids was estimated by the following equation: 

AID (%) = 100—[(Ti _(diet)_ × AA _(ileum)_)/(TI _(ileum)_ × AA_(diet)_) × 100].

Ti (diet): the titanium dioxide concentration in the diet. 

Ti (ileal): the titanium dioxide concentration in ileal digesta. 

AA (ileal): the concentration of the test AA in the ileal digesta sample.

AA (diet): the concentration of the test AA in the diet.

### 2.4. Sample Collection and Laboratory Analyses

At the end of the feeding period (35 days), blood samples were randomly collected from five birds per treatment after slaughter into rubber stoppers sterilized tubes. Samples were left to coagulate at 4 °C and centrifuged at 3500 rpm for 15 min to obtain serum, and the serum samples were retained in Eppendorf tubes at –20 °C until they were analyzed. Samples from different parts of the small intestine (duodenum, jejunum, ileum) were taken for histological examination.

An enzymatic method was used to determine total serum cholesterol with a single aqueous reagent using colorimetric diagnostic kits of spectrum-bioscience (Egyptian Company for Biotechnology, Cairo, Egypt) following the methods of Allain et al. [29]. A peroxidase-coupled method was used for the colorimetric determination of serum triglycerides following the practices of McGowan et al. [30]. The enzymatic colorimetric method was used to determine the serum level of high density lipoprotein (HDL)-C following the procedures of Vassault et al. [31]. The Iranian formula of low density lipoprotein (LDL)-C = total cholesterol (TC)/1.19 + triglycerides (TG)/1.9 − HDL/1.1 − 38 was used for LDL-C calculation. Chicken ELISA kits of MyBioSource Co. of CAT.NO. MBS012469, MBS701683, and of ABCAM Co. of CAT. NO. AB157691 were used to determine the serum levels of alkaline phosphatase, interleukin 10, and IgM, respectively. Meanwhile, a sandwich enzyme-linked immunosorbent assay (ELISA) kit manufactured by Life Span Biosciences, Inc. of CAT.NO.LS-F9287 was used for determining serum complement 3 levels by following the manufacturer’s instructions.

### 2.5. Histological Examination of the Small Intestine

The whole small intestinal tract was sampled for histomorphological examination. Two-cm tissue samples were taken from the duodenum, jejunum, and ileum, according to the method of Giannenas et al. [32]. The tissue samples were briefly preserved in 10% neutral buffered formaldehyde (NBF) for 72 h, then processed for dehydration and clearing, and embedded in wax. Histological study was performed on 5-µm thick transverse sections (cut by a microtome), fixed on slides, and stained with hematoxylin and eosin [33] and acidic mucus containing goblet cells were identified using periodic acid-Schiff (PAS) staining. The mucosal and muscular layer of the duodenum, jejunum and ileum were examined using a digital camera (Canon) connected to a light microscope (Zeiss). Camera microscope AmScope® software (AmScope digital cameraattached Ceti England microscope) was used for morphometric analysis as follows: villus height was measured from the tip (with a lamina propria) of the villus to the base (villus–crypt junction), crypt depth was measured from the villus–crypt intersection to the distal limit of the crypt, and the thickness of the tunica muscularis was defined as the distance between the lamina muscularis mucosae internally and the tunica serosa externally. ImageJ was used to calculate the number of goblet cells in PAS stained sections per unit of epithelial area (mm^2^) and individual goblet cell areas (μm^2^).

### 2.6. Statistical Analysis

Data were analyzed with a one-way analysis of variance (ANOVA) using the general linear model procedure in SPSS sofware (SPSS Inc., Chicago, Illinois, USA) after Shapiro–Wilk’s test was used to verify the normality and Levene’s test was used to verify homogeneity of variance components between experimental treatments. Tukey’s test was used to compare the differences between the means at 5% probability. Variation in the data was expressed as pooled standard error of mean (SEM), and the significance level was set at *p* < 0.05.

## 3. Results

### 3.1. Growth Performance

Glycerol monolaurate supplementation had no significant effect on BW, BWG, feed intake (FI), FCR, PER, and RGR all over the experimental periods compared to the control group (*p* > 0.05) (Table 2).

### 3.2. Apparent Ileal Digestibility Coefficient (AID%) of Amino Acids

As shown in Table 3, The AID% of leucine was increased in the GML1 diet and decreased by increasing glycerol monolaurate level (*p* = 0.003). The AID% of arginine was significantly reduced in the GML1 diet (*p* < 0.05). The AID% of threonine, lysine, methionine, tryptophan, valine, and isoleucine were not significantly different between the control and other supplemented groups (*p* > 0.05).

### 3.3. Morphometric Measures of the Small Intestine

**Figure 1:**
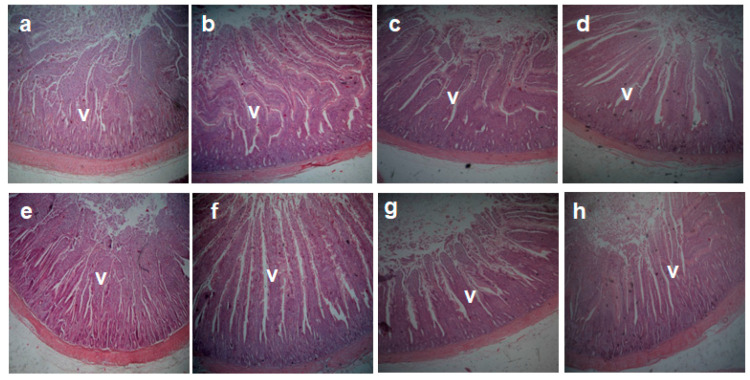
A representative photomicrograph of 40× magnification H&E stained small intestine sections of the broiler chickens. (**a**–**d**) Duodenal sections from control group, GML1, GML3, and GML5 groups, respectively, showing increase in villus (V) length in GML1, GML3 groups and decrease in the GML5 group. (**e**–**h**) Jejunal sections from the control group, GML1, GML3, and GML5 groups, respectively, showing increase in villus (V) length in the GML1 group compared to the control group.

**Figure 2:**
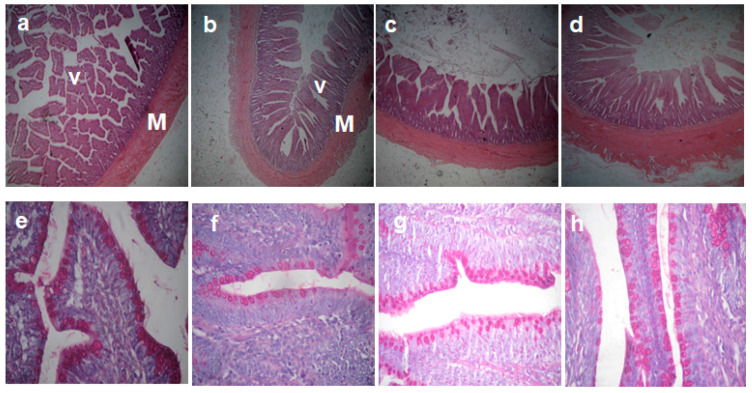
A representative photomicrograph of 40× magnification H&E stained small intestine sections of the broiler chickens. (**a**–**d**) Ileal sections from the control group, GML1, GML3, and GML5 groups, respectively, showing no significant difference in the villous height (V), crypt depth and muscle thickness (M) between different groups. (**e**–**h**) Goblet cells (arrow) in the ileum from the control group, GML1, GML3, and GML5 groups, respectively, increased in the GML3 group compared to the control group.

The morphometric measurements of the different parts of the small intestine of birds fed on the experimental diets are shown in Table 4 and Figure 1 and Figure 2. The duodenal villous height was increased in the GML1 and GML3 groups and decreased in the GML5 group (*p* = 0.00). The goblet cell count (GCC) in the duodenum was raised in the GML3 and GML5 groups compared to the GML1 group (*p* = 0.009), but it was not significantly different from the control group. The jejunal muscle thickness and villous height were increased in the GML1 group compared to the control group (*p* < 0.05). The jejunal crypt depth was decreased in the GML3 and GML5 groups (*p* = 0.00). The GCC in the jejunum was not significantly different in all glycerol monolaurate-supplemented groups compared to the control group. The goblet cell count in the ileum was increased in the GML3 group compared to the control group (*p* = 0.003). The mucosal thickness, villous height, and crypt depth of the ileum were not significantly different among the groups (*p* > 0.05).

### 3.4. Lipid Profile and the Level of Alkaline Phosphatase “ALP”

Supplementation of GML increased the serum level of HDL-cholesterol compared to GML0 (*p* = 0.01). GML5 diet increased the serum level of triglycerides in comparison with GML1 and GML3 diets (*p* = 0.009), but its level was not significantly different in all GML-supplemented groups compared to GML0 (*p* > *0.05*). Supplementation of GML had no significant effect on the serum levels of ALP, total cholesterol, and LDL-cholesterol (*p* > 0.05) (Table 5).

### 3.5. Immune Status

The GML5 diet increased the serum levels of IgM and interleukin 10 compared to the control group (*p* < 0.05). Supplementation of GML had no significant influence (*p* > 0.05) on the serum level of complement 3 (Table 6).

## 4. Discussion

With the recent ban and restrictions of using antibiotics as growth promoters, many feed supplements are widely added in poultry feeds as alternative growth promoters, such as prebiotics, phytobiotics, probiotics, organic acids, and enzymes [24,34,35,36,37,38]. Recently, MCFAs have received more consideration because of their potential antimicrobial effects. Growth performance can be improved by 4% and 12% for increased BW and FCR, respectively, due to improved gut health and environment by MCFAs [39]. MCFAs are usually documented as safe (GRAS) by the Food and Drug Administration [40]. Dietary MCFAs have been reported to enhance the growth performance and intestinal histomorphology and decrease the invasion of the intestinal pathogen and mortality in broiler chickens [41,42] and Japanese quail [43]. Many studies have shown that the effectiveness of MCFAs in stimulating bird growth will produce a final result to improve the gastrointestinal ecology, with the ensuing intestinal environment, intestinal mucosal integrity, the digestive and immune status of the gut, and the broiler chickens health [44,45,46]. Other studies have shown no improvement in broiler chickens’ performance when the birds fed on MCFAs-complemented diets are compared to control birds and antibiotics-fed birds [47,48].

The current results show that dietary supplementation of graded levels of glycerol monolaurate had no improving effect on the bird’s growth throughout the experimental period, which may be due to the insignificant effect of GML supplementation on the AID% of amino acids except for the increased AID% of leucine in the GML1 group and the decreased AID% of arginine in the GML1 group. Moreover, GML supplementation did not affect the FI and feed utilization, where feed intake is an essential feature guiding the broiler’s growth rate [49]. Similar results were informed in quail chicks fed MCFAs supplemented diets by levels of 1, 2, and 4 g/kg at different experiment stages [43]. Hejdysz et al. [50] showed no significant improvement of the bodyweight gains by medium-chain fatty acids supplementation in chickens. Liu et al. [51] observed no significant difference in growth performance during the starter and grower stage, but during the finisher period, the average daily feed intake and BW were increased by supplementation of 300, 450, and 600 mg/kg medium-chain α-monoglycerides. They attributed the improved body weight to the increased feed intake caused by the supplementation. Lipiński et al. [52] reported enhanced turkey growth by dietary supplementation with MCFA glycerides and a herbal additive. Devi and Kim [53] showed a significant elevation in the average daily gain and feed efficiency ratio of pigs caused by MCFAs supplementation alone or with probiotics. Mabayo et al. [54] informed that MCTs-supplementation of the chick diet improved body protein utilization and weight gain while controlling fat deposition compared to the LCTs-supplemented diet. Allee et al. [55] reported no significant changes in WG, FI, and feed efficiency of pigs fed 10% MCTs supplemented diet compared to those fed on diets complemented with tallow, pig fat, or corn oil at the same level. Furthermore, the results of Baltić et al. [56] showed that broiler diets supplemented with MCFAs affected the broiler performance positively. Decuypere and Dierick [57] attributed the improved growth performance to the antibacterial effects of the MCFAs, especially against pathogenic bacteria. Others have related the performance effects to the effect of MCFAs on the epithelial function in the upper small intestine, either directly or indirectly by increasing the absorptive surface, increasing uptake and producing a higher nutrient utilization for growth. Intestinal cells can immediately utilize MCFAs to produce energy and thus support the intestinal tissue integrity in post-weaning piglets [15]. Rats fed on MCTs-supplemented diets showed improved intestinal morphology indicated by mucosa augmentation, longer intestinal villi, shorter crypts, increased phospholipid/protein ratio in the lipids’ jejunal mucosal, and increased membrane-bound enzymes activity [58].

The small intestine has a pivotal role in nutrient digestion and absorption in broiler chickens. Improving intestinal development increases the utilization of nutrients by the broiler, leading to improved growth performance [41]. Although the current study results showed an optimistic effect of glycerol monolaurate supplementation on the morphometric measures (muscle thickness and villous height (VH)) of the different parts of the intestine, especially the GML1 group, this improvement was not reflected on the birds’ growth or nutrient digestibility. Several studies showed that dietary supplementation of MCFA increased the VH, villous width (VW), and the absorptive area of the small intestine of broiler chicks at 14 days old [59,60,61,62,63]. Leeson et al. [64] and Panda et al. [65] reported improved duodenal VH and crypt depth (CD) by MCFAs supplementation in broilers’ diet that could be helpful for intestinal development in young birds. High duodenal and ileal villus heights by MCFAs supplementation in birds’ diets were also documented by [56,66]. Increasing villus length can increase enzyme production and improve digestion by increasing effective absorbance and enhancing nutrient absorption [67]. MCFA and related glycerides can be directly taken up by enterocytes to produce energy in the small intestine and improve gut growth and integrity in farm animals [68]. Liu et al. [51] reported an increase in the jejunal villus length and VH: CD ratio in the duodenum and ileum by dietary GML. Zhao, et al. [69] reported improved Intestinal morphology by GML supplementation indicated by increased VH and VH: CD ratio. Liu, et al. [70] reported improved growth, muscle amino acids, and intestinal morphology in broilers by GML supplementation (300, 450, and 600 mg/kg) mostly by influencing the gut microbiota function and community. Pan and Yu [71] demonstrated that short chain fatty acids (SCFAs) can act as energy source for intestinal epithelial cells and enhance the growth and proliferation of intestinal cells. Thus, one possible clarification for improving gut morphology by GML may be derived from the high SCFAs levels in the gastrointestinal tract. Furthermore, increased SCFAs may improve the intestinal health and may assist the host in maintaining mucosal barrier integrity [70,72]. Mo, et al. [73] showed that dietary GML improved the intestinal integrity and gut barrier function in mice.

Concerning the effect of glycerol monolaurate supplementation on the lipid profile of broiler chickens, GML supplementation increased the serum level of HDL-cholesterol and the GML5 group displayed higher serum levels of triglycerides than GML1 and GML3 groups. These results agree with the results of Liu et al. [51], who demonstrated increased serum HDL-cholesterol in birds fed with 300 and 450 mg/kg medium-chain a-monoglycerides. Modification of the serum lipid profile showed that dietary medium chain glycerides could efficiently enhance the fat metabolism in broiler chickens. MCFA and the related glycerides were associated with increased carriage of extra cholesterol, leading to lower serum cholesterol levels [74,75]. MCFA supplementation in broiler food reduced LDL cholesterol levels and increased HDL-C content [76]. Baltić et al. [56] showed increased serum triglyceride level and no significant effect on HDL-C level in broilers with MCFAs supplementation. The increased triglyceride level caused by MCFAs supplementation observed in this study is in line with the results of [77]. However, the MCFAs’ effects on cholesterol and triglyceride appeared to be conflicting, as cholesterol and triglyceride secretion is controlled in an organized way [8]. A possible justification could be that increased MCFAs motivated insulin secretion and stimulated anabolic-related processes. Consequently, an increase in de novo fatty acid synthesis may cause a rise in the production of triglycerides [77]. Saeidi et al. [43] reported that quail chicks fed on MCFA-supplemented diet showed decreased serum levels of TC, TG, LDL-C, and increased HDL-C. Zhao *et al.* [69] reported an optimistic effect of GML supplementation on the lipid profile of laying hens indicated by reducing the the serum TG and TC levels and lowering the abdominal fat. 

Concerning the Effect of GML on broiler chickens’ immune status, the GML5 diet significantly raised the serum levels of IL10 and IgM. MCFAs bind the orphan receptor GPR84, a signal protein that is mostly expressed in immune cells. GPR84 activated in monocytes and macrophages that boosted lipopolysaccharide production (LPS) and stimulated IL-12 p40, which suggests a mechanism that fixes free fatty acids with immune responses [78]. Even though MCFAs are freely absorbed in the upper intestine, the effect of MCFAs on ILs was studied with colonic cells. MCTs-fed rats showed a rise in IL-6 expression and immunoglobulin A (IgA) secretion after bacterial LPS injection [79].

Moreover, MCTs supplementation decreased the LPS-induced expression of proinflammatory chemokines and cytokines and increased anti-inflammatory and immune-modulating cytokine IL-10 in the ileum and Peyer’s patches. IgA secretion and modification of cytokine release after LPS injection were proposed to intermediate optimistic effects on animal intestinal health [79]. Dietary MCFAs can resist the microbial activity of Salmonella and Escherichia coli [80,81]. Dietary MCFAs may boost immune function, antibiotic alternatives in poultry nutrition, and decrease the hazard of antimicrobial resistance [39].

## 5. Conclusions

Glycerol monolaurate can supplement broiler chicken diets up to 5 g kg^−1^ in order to enhance the immune status and intestinal histomorphology of birds with no improving effect on the growth performance or amino acid digestibility.

## Figures and Tables

**Table 1:** The proximate chemical composition of the basal diet as fed basis (%).

Ingredients	Unit	Starter	Grower	Finisher
Corn 7.5% cp	%	53.02	58	62.50
Soybean meal 47% cp	%	37	32	26.00
Corn gluten meal 60% cp	%	3.5	3.2	4.50
Oil (soya)	%	2	3	3.50
Dicalcium phosphate 18%	%	2	1.7	1.45
Calcium carbonate	%	0.7	0.5	0.50
Sodium bicarbonate	%	0.35	0.33	0.32
Dl methionine 99%	%	0.36	0.28	0.30
Broiler premix *	%	0.3	0.3	0.30
L-LYSINE HCL 98%	%	0.3	0.3	0.28
Salt	%	0.15	0.11	0.13
Antimycotoxin	%	0.1	0.1	0.10
Choline 60 veg	%	0.07	0.07	0.07
L-THREONINE 98.5%	%	0.1	0.1	0.06
Enzyme Phytase	%	0.005	0.005	0.005
Chemical analysis **				
Moisture	%	11.27	11.23	11.17
ME poultry. (kcal/kg)	Kcal/kg	3007.24	3103.42	3202.13
Crude protein analysis	%	23.70	21.55	20.13
Crude protein %	%	24.22	21.90	20.42
Lysine g	g/kg	14.56	13.20	11.59
Methionine g	g/kg	7.25	6.18	6.14
Methionine + cystine g	g/kg	10.90	9.58	9.40
Threonine g	g/kg	9.99	9.13	8.12
Tryptophan g	g/kg	2.79	2.49	2.19
Arginine g	g/kg	15.32	13.72	12.15
Valine g	g/kg	11.32	10.27	9.58
Crude Fat %	%	4.98	6.04	6.68
C18:2 linoleic ac. %	%	2.32	2.90	3.22
Ash %	%	6.35	5.53	5.01
Calcium g	g/kg	9.47	8.95	8.21
Av. Phosphorus	g/kg	4.97	4.47	4.03
Cl g	g/kg	2.63	2.32	2.35
Sodium g	g/kg	1.85	1.62	1.67
Potassium g	g/kg	8.84	8.05	7.06
Manganese mg	mg/kg	102.81	99.69	96.34
DEB	meq/kg	247.64	223.99	197.65
Crude fiber	%	3.426	3.23	2.98
A.D.F.	%	4.69	4.43	4.10
N.D.F.	%	10.33	10.22	10.01
Starch %	%	35.09	38.27	41.20
Total Sugar %	%	3.88	3.54	3.13
Vit A	kU.I	9.55	9.55	9.55
Vit D3	kU.I	4.66	2.66	2.66
Vit E	mg/kg	19.14	19.14	19.14
Choline chloride	mg/kg	1732.12	1625.40	1534.00
Choline chloride equivalent	mg/kg	420	420	420.00

* Premix per kg of diet: vitamin A, 1500 IU; vitamin D3, 200 IU; vitamin E, 10 mg; vitamin K3, 0.5 mg; thiamine, 1.8 mg; riboflavin, 3.6 mg; pantothenic acid, 10 mg; folicacid, 0.55 mg; pyridoxine, 3.5 mg; niacin, 35 mg; cobalamin, 0.01 mg; biotin, 0.15 mg; Fe, 80 mg; Cu, 8 mg; Mn, 60 mg; Zn, 40 mg; I, 0.35 mg; Se, 0.15 mg. ** According to (Aviagen, 2014). Cp: crude protein, ME: metabolizable energy, DEB: dietary electrolyte balance. A.D.F.: Acid detergent fiber, N.D.F.: Neutral detergent fiber.

**Table 2:** The effect of glycerol monolaurate supplementation on the growth performance of broiler chickens.

Parameters	GML0	GML1	GML3	GML5	SEM	*p* Value
Initial weight (g)	77.00	77.41	75.45	77.62	0.40	0.67
Starter period						
BW(g)	222.66	219.70	220.87	223.04	2.02	0.96
BWG(g)	145.66	142.29	145.41	145.41	2.39	0.95
FI (g)	187.41	189.20	188.45	187.33	2.26	0.98
FCR	1.28	1.33	1.30	1.29	0.01	0.84
Grower period						
BW(g)	876.91	858.00	851.29	856.50	14.80	0.92
BWG(g)	654.25	638.29	630.41	633.45	10.66	0.92
FI (g)	994.08	923.54	957.37	955.37	9.52	0.51
FCR	1.527	1.445	1.519	1.512	0.01	0.55
Finisher period						
BW(g)	1932.91	1851.00	1879.45	1909.37	31.37	0.93
BWG(g)	1056.00	993.00	1028.16	1052.87	29.23	0.92
FI(g)	2074.25	1978.50	2030.91	2124.00	22.27	0.81
FCR	1.075	1.073	1.08	1.12	0.017	0.93
Overall performance						
BW(g)	1932.91	1851.00	1879.45	1909.37	31.37	0.93
BWG(g)	1855.91	1773.58	1804.00	1831.75	29.001	0.93
FI (g)	3255.75	3091.25	3176.75	3266.70	42.87	0.78
FCR	1.75	1.75	1.76	1.79	0.025	0.98
PER	2.74	2.75	2.73	2.72	0.03	0.99
RGR	184.61	183.79	184.45	184.32	0.19	0.82

Means within the same row carrying different superscripts are significantly different at (*p* < 0.05). BW: body weight, BWG: body weight gain, FI: feed intake, FCR: feed conversion ratio, PER: protein efficiency ratio, RGR: relative growth rate. GML0, GML1, GML3, and GML5: basal diets supplemented with 0, 0.1 or 0.3 or 0.5% glycerol monolaurate, respectively.

**Table 3:** The effect of glycerol monolaurate supplementation on the apparent ileal digestibility coefficient (AID%) of amino acids.

Parameters	GML0	GML1	GML3	GML5	SEM	*p* Value
Lysine	89.12	88.82	88.95	88.87	0.08	0.16
Methionine	87.54	87.7	87.21	87.46	0.06	0.12
Threonine	85.71	85.46	85.22	85.09	0.12	0.07
Tryptophan	87.67	77.63	87.44	86.99	0.18	0.47
Arginine	90.45 ^a^	89.95 ^b^	90.20 ^ab^	90.37 ^ab^	0.03	0.04
Valine	86.01	85.59	85.69	85.54	0.05	0.07
Leucine	90.68 ^b^	91.01 ^a^	90.42 ^bc^	90.31^c^	0.05	0.003
Isoleucine	86.10	86.34	85.98	86.24	0.05	0.22

^a,b,c^ Means within the same row carrying different superscripts are significantly different at (*p* < 0.05). TC: total cholesterol, TG: triglycerides, HDL: high density lipoprotein, LDL: low density lipoprotein. GML0, GML1, GML3, and GML5: basal diets supplemented with 0, 0.1 or 0.3 or 0.5% glycerol monolaurate, respectively.

**Table 4:** The effect of glycerol monolaurate supplementation on the morphometric measures (µm) of the small intestine of broiler chickens.

Parameters	GML0	GML1	GML3	GML5	SEM	*p* Value
Duodenum						
Muscle thickness	121.80	125.61	114.78	114.01	9.30	0.78
Crypt depth	247.98	243.59	205.93	186.05	13.78	0.19
Villus height	582.39 ^c^	1111.66 ^a^	796.03 ^b^	716.12 ^bc^	56.01	0.00
Goblet cell count	25 ^ab^	22.66 ^b^	32.33 ^a^	31 ^a^	1.32	0.009
Jejunum						
Muscle thickness	117.06 ^b^	183.72 ^a^	142.94 ^ab^	106.18 ^b^	26.90	0.01
Crypt depth	252.83 ^a^	223.38 ^a^	128.83 ^b^	158.91 ^b^	11.60	0.00
Villus height	672.81 ^b^	1067.44 ^a^	770.84 ^b^	715.82 ^b^	45.57	0.00
Goblet cell count	40.66	35	44.66	39.33	1.58	0.05
Ileum						
Muscle thickness	219.02	246.56	175.79	235.28	17.93	0.55
Crypt depth	117.06	124.81	110.02	112.75	7.59	0.45
Villus height	323.87	323.35	363.15	371.23	29.81	0.18
Goblet cell count	70 ^ab^	56.33 ^b^	80 ^a^	55.66 ^b^	2.57 ^b^	0.003

^a,b,c^ Means within the same row carrying different superscripts are significantly different at (*p* < 0.05). GML0, GML1, GML3, and GML5: basal diets supplemented with 0, 0.1 or 0.3 or 0.5% glycerol monolaurate, respectively.

**Table 5:** The effect of glycerol monolaurate supplementation on the lipid profile and the level of alkaline phosphatase (ALP) of broiler chickens.

Parameters	GML0	GML1	GML3	GML5	SEM	*p* Value
TC (mg/dL)	218.36	223.80	229.53	229.58	2.39	0.43
TG (mg/dL)	143.08 ^ab^	125.92 ^b^	132.01 ^b^	195.6 ^a^	5.63	0.009
HDL(mg/dL)	34.66 ^b^	51.23 ^a^	72.05 ^a^	72.28 ^a^	3.34	0.011
LDL(mg/dL)	161.07	174.57	192.32	149.70	8.70	0.16
ALP (U/L)	39.49	39.34	41.17	51.95	6.35	0.32

^a,b^ Means within the same row carrying different superscripts are significantly different at (*p* < 0.05). TC: total cholesterol, TG: triglycerides, HDL: high density lipoprotein, LDL: Low density lipoprotein. GML0, GML1, GML3, and GML5: basal diets supplemented with 0, 0.1 or 0.3 or 0.5% glycerol monolaurate, respectively.

**Table 6:** The effect of of glycerol monolaurate supplementation on the immune status of broiler chickens.

Parameters	GML0	GML1	GML3	GML5	SEM	*p* Value
IgM (mg/dL)	42.65 ^b^	72.13 ^b^	75.01 ^b^	132.01 ^a^	10.89	0.001
C3 (mg/dL)	67.00	64.87	89.27	80.24	5.55	0.28
IL10 (pg/mL)	0.12 ^b^	0.17 ^b^	0.25 ^ab^	0.30 ^a^	0.01	0.004

^a,b^ Means within the same row carrying different superscripts are significantly different at (*p* < 0.05). GML0, GML1, GML3, and GML5: basal diets supplemented with 0, 0.1 or 0.3 or 0.5% glycerol monolaurate, respectively.

## Data Availability

Data sharing not applicable.
